# APOA1BP and natural killer cells as diagnostic and pathophysiological biomarkers in schizophrenia-associated cognitive impairment: A multi-cohort bioinformatics analysis

**DOI:** 10.1097/MD.0000000000046649

**Published:** 2025-12-12

**Authors:** Xu You, Hongming Liang, Huabin Yang, Rongzong Shi

**Affiliations:** a Department of Psychiatry, The Second People’s Hospital of Honghe Hani and Yi Autonomous Prefecture, Honghe, Yunnan, China.

**Keywords:** APOA1BP, biomarkers, cognitive impairment, natural killer cells, schizophrenia, transcriptomics

## Abstract

Schizophrenia (SZ) is frequently accompanied by cognitive impairment, yet validated molecular biomarkers remain limited. This study aimed to identify immune-related biomarkers, particularly APOA1BP and natural killer (NK) cells, through integrative bioinformatics analyses. Three gene expression omnibus transcriptomic datasets (GSE93987, GSE87610, GSE73129) were analyzed. Differentially expressed genes (DEGs) were identified and evaluated using Weighted Gene co-expression Network Analysis (WGCNA) and LASSO regression. Immune cell infiltration was assessed by single-sample gene set enrichment analysis (ssGSEA). Diagnostic performance of candidate genes was validated in independent cohorts using receiver operating characteristic analysis. Seventy DEGs were identified, mainly involved in extracellular matrix and cell adhesion. Four candidate genes (APOA1BP, C12orf57, MRPL46, ZDHHC11) were highlighted, with APOA1BP consistently downregulated in SZ. ROC analysis demonstrated diagnostic potential (AUC up to 0.76), though performance varied across tissues. NK cell infiltration was significantly elevated in SZ and negatively correlated with APOA1BP and MRPL46. Validation confirmed APOA1BP as the most robust biomarker in DLPFC-derived datasets, while olfactory epithelial-derived samples showed limited significance, likely reflecting tissue-specific heterogeneity. This multi-cohort bioinformatics analysis identifies APOA1BP and NK cells as promising biomarkers in SZ-associated cognitive impairment. While findings are correlative, they suggest that immune-metabolic interactions may contribute to SZ pathophysiology. Future research should validate these biomarkers in larger, clinically annotated cohorts and explore their mechanistic and therapeutic potential.

## 1. Introduction

Schizophrenia (SZ) is a severe psychiatric disorder with a strong genetic predisposition,^[1]^ that affects approximately 1% of the global population during their lifetime.^[2]^ According to the global burden of disease study, approximately 23.6 million people globally are diagnosed with schizophrenia, highlighting its significant societal and healthcare impact.^[3]^ Clinically, SZ is characterized by a spectrum of symptoms, including hallucinations, delusions, and disturbances in thought processes and behavior, which significantly impair daily functioning and often lead to chronic disability.^[4]^ In addition to positive symptoms, SZ is marked by negative symptoms and cognitive deficits, the latter affecting over 80% of patients and profoundly influencing their quality of life.^[5]^ These cognitive impairments have been linked to dysfunction in key brain regions such as the dorsolateral prefrontal cortex (DLPFC), medial prefrontal cortex, and parietal lobe,^[6]^ with studies highlighting the role of pyramidal neurons in the DLPFC in cognitive dysfunction.^[7,8]^

Notwithstanding comprehensive investigation, the identification of SZ continues to depend on clinical assessment, given that no conclusive pathological or molecular diagnostic indicators are accessible.^[9–11]^ Complicating the diagnostic process even more, SZ frequently coexists with or exhibits overlapping clinical characteristics with other psychiatric disorders, such as bipolar disorder and depression, indicating shared genetic and biological foundations.^[12]^ Moreover, SZ typically follows a prolonged course, with recurrent episodes and potential symptom exacerbation, contributing to its complex and multifaceted nature.^[13]^ Recent studies have highlighted the role of immune dysregulation in SZ pathogenesis.^[14,15]^ Evidence indicates a disparity in inflammatory cytokine signaling, characterized by diminished secretion of type 1 helper T cytokines and heightened levels of type 2 helper T cytokines, highlighting a disturbed interaction between the immune system and the central nervous system.^[16]^ Immune-related mechanisms have also been implicated in cognitive dysfunction, particularly within pyramidal neurons of the DLPFC, although the precise molecular pathways remain unclear.^[17,18]^

Given the challenges in diagnosing SZ and understanding its cognitive and immune-related pathophysiology, this study aimed to identify immune-related molecular markers associated with SZ using an integrative bioinformatics approach. By leveraging publicly available gene expression datasets from the GEO database, we analyzed the transcriptomic profiles from the DLPFC and olfactory epithelial (OE) cells of patients with SZ. Key genes were identified using differential expression analysis, WGCNA, and LASSO regression. Furthermore, we utilized ssGSEA to explore immune cell infiltration patterns and their relationships with identified key genes and sought to bridge the gap in understanding the molecular mechanisms underlying SZ by elucidating the immune-related processes contributing to cognitive dysfunction. Identifying immune biomarkers may not only enhance diagnostic precision, but also pave the way for novel therapeutic strategies targeting immune pathways in SZ.

## 2. Material and methods

### 2.1. Accessing and obtaining the data

For this study, we retrieved datasets from the Gene Expression Omnibus (GEO) database that met the specified conditions using the keyword “Schizophrenia” (https://www.ncbi.nlm.nih.gov/). This study used only publicly available, de-identified transcriptomic datasets from the GEO database. As no experiments involving human participants or animals were conducted, institutional review board approval and informed consent were not required, and the inclusion criteria for dataset selection were studies utilizing expression profiling by array and datasets derived exclusively from Homo sapiens. Based on these criteria, 3 datasets were selected: GSE93987 (based on the GPL13158 platform of Affymetrix HT HG-U133 + PM Array Plate), GSE87610 (based on the GPL13667 platform of Affymetrix Human Genome U219 Array), and GSE73129 (based on the GPL570 platform of Affymetrix Human Genome U133 Plus 2.0 Array). The GSE93987 dataset served as the training cohort and contained gene expression profiles from 3 layers of pyramidal neurons in DLPFC samples. It consists of 2 subcohorts, GSE93987-1 and GSE93987-2, representing distinct sampling positions within the same brain region. 2 validation datasets were used: GSE87610, featuring gene expression from pyramidal neurons in the postmortem DLPFC, and GSE73129, profiling neuronal layers of the OE from living SZ patients. (Details were presented in Table 1.)In addition to the general inclusion criteria, dataset selection was guided by biological relevance. GSE93987, derived from DLPFC pyramidal neurons, was chosen as the training cohort because the DLPFC is strongly implicated in WM and executive dysfunction in SZ. To minimize variability, GSE87610 – also obtained from postmortem DLPFC neurons – was included as a validation cohort to confirm reproducibility within the same brain region. To further evaluate potential translational relevance, we incorporated GSE73129, which profiles neuronal layers of the OE from living patients with SZ. The OE is increasingly studied as a peripherally accessible neuronal tissue, offering opportunities for biomarker development beyond postmortem brain tissue. This stepwise selection enabled us to assess both brain region specificity and peripheral exploratory potential.

**Table 1:** Basic information of training and validating datasets in the study.

Group	GSE ID	Tissues	Samples	Platform	Last update date
Training cohort	GSE93987-1	Layer 3 of DLPFC	36 SZ and 35 HC	GPL13158	April 20, 2018
	GSE93987-2	Layer 3 of DLPFC	17 SZ and 17 HC	GPL13158	April 20, 2018
Validating cohort	GSE87610	Layer 3 of DLPFC	34 SZ and 36 HC	GPL13667	December 22, 2020
	GSE73129	Neuronal layers of OE	20 SZ and 20 HC	GPL570	March 25, 2019

SZ = schizophrenia.

### 2.2. Data processing and DEG identification

Gene expression data were normalized and standardized using the “sva” package based on matrices and annotation files from the GEO database. Differentially expressed genes (DEGs) between patients with SZ and controls were identified using the “limma” package, with selection criteria set at *P* <.05, and |log2FC| >0.5.

### 2.3. Enrichment analysis and WGCNA construction

For a better understanding of the biological processes and signal pathways linked with SZ DEGs, gene ontology (GO) and Kyoto encyclopedia of genes and genomes (KEGG) enrichment analyses were performed on DEGs using the “clusterProfiler” package in R, with significance set at adjusted *P* <.05. WGCNA was implemented to construct biological networks using the “WGCNA” package, followed by module-trait association analysis identifying SZ-related hub modules.^[19]^ The process included determining the soft threshold power(β) using the pickSoftThreshold function to meet scale-free topology criteria, clustering genes to dynamically detect and merge similar gene modules, and calculating module-phenotype correlations to identify modules strongly linked to SZ. We evaluated module relevance through gene significance (GS = 0.2) and module membership (MM = 0.6) to pinpoint SZ-related hub genes.

### 2.4. Identification of key genes and LASSO regression analysis

DEGs were cross-referenced with hub genes from SZ-associated modules using the Venn online tool (http://bioinformatics.psb.ugent.be/webtools/Venn/) to identify the intersecting genes. Key biomarkers were further refined using LASSO regression, which minimizes estimation variance in high-dimensional datasets^[20]^ and ultimately identified hub genes with network topological centrality.^[21]^ The expression data of intersecting genes were analyzed with the “glmnet”package in R to identify the most representative SZ-related biomarkers.

### 2.5. Validation of key genes in the training cohort

Key genes identified in the GSE93987 training cohort were validated using the “ggpubr”package to assess their differential expression (upregulation or downregulation), with a significance threshold of *P* <.05. The diagnostic potential of these genes was further evaluated using the “pROC”package to analyze their receiver operating characteristic (ROC) curves.

### 2.6. ssGSEA analysis of key genes

ssGSEA was performed to evaluate the immune cell landscape and functionality in patients with SZ. The “gsva”package was used to calculate hallmark gene set scores, enabling the assessment of differential immune cell enrichment between SZ patients and healthy controls. Additionally, correlations between key genes and immune cell populations have been analyzed to explore their immunological relevance.^[22]^

### 2.7. Validation of differential expression and diagnostic value in the testing cohort

The differential expression and diagnostic significance of targeted genes were validated in the testing cohort using the “ggpubr” and “pROC”packages. This analysis aimed to confirm whether the findings from the training cohort were consistent in independent datasets, thereby reinforcing the reliability and diagnostic potential of the identified genes.

## 3. Results

### 3.1. DEGs screening

**Figure 1.**
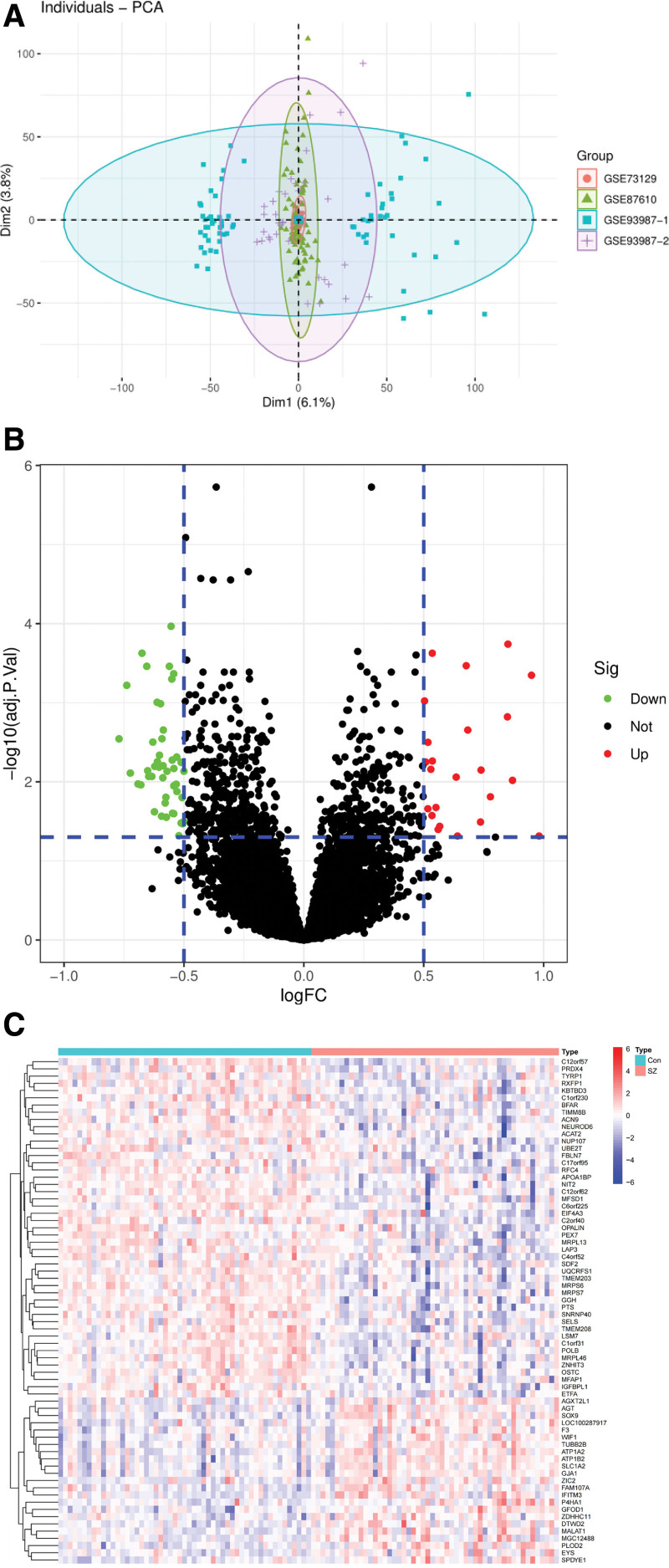
(A) Consistency of gene expression levels across datasets GSE93987, GSE87610, and GSE73129, demonstrating effective mitigation of batch effects. (B) Volcano plot illustrated the 70 DEGs identified in the GSE93987 dataset. (C) Heatmap showed the DEGs ranked by *P*-value. DEGs = differentially expressed genes.

As shown in (Fig. 1A), principal component analysis was utilized to assess the consistency of gene expression levels across datasets GSE93987, GSE87610, and GSE73129. The principal component analysis results indicate clear clustering of samples, demonstrating the effective removal of batch effects and confirming data comparability across different datasets. Using a threshold of *P* <.05 and |Log2FC| >0.5, 70 DEGs were identified from the combined GSE93987-1 and GSE93987-2 datasets, comprising 23 upregulated and 47 downregulated genes. A volcano plot was generated to visualize these DEGs (Fig. 1B), and a heatmap was used to highlight the DEGs ranked by P-values (Fig. 1C).

### 3.2. Functional enrichment analysis of DEGs

The results of GO and KEGG enrichment analysis of DEGs (top 5) were shown in Tables 2 and 3, respectively.

**Table 2:** GO enrichment analysis of DEGs associated with SZ.

GO term	ID	Description	*P*-value
BP	GO:0086064	cell communication by electrical coupling involved in cardiac conduction	5.13 × 10^−05^
BP	GO:0010644	cell communication by electrical coupling	.0001
BP	GO:0021537	telencephalon development	.0001
BP	GO:1905332	positive regulation of morphogenesis of an epithelium	.0001
BP	GO:0061337	cardiac conduction	.0001
CC	GO:0000313	organellar ribosome	.0001
CC	GO:0005761	mitochondrial ribosome	.0001
CC	GO:0005684	U2-type spliceosomal complex	.0001
CC	GO:0098798	mitochondrial protein-containing complex	.0002
CC	GO:0005890	sodium:potassium-exchanging ATPase complex	.0005
MF	GO:0031418	L-ascorbic acid binding	.0016
MF	GO:0003735	structural constituent of ribosome	.0020
MF	GO:1901682	sulfur compound transmembrane transporter activity	.0138
MF	GO:0016706	2-oxoglutarate-dependent dioxygenase activity	.0142
MF	GO:0016705	oxidoreductase activity, acting on paired donors, with incorporation or reduction of molecular oxygen	.0169

GO analysis of DEGs genes of SZ (Top 5).

BP = biological process, CC = cell composition, DEGs = differentially expressed genes, GO = gene ontology, MF = molecular function, SZ = schizophrenia.

**Table 3:** KEGG enrichment analysis of DEGs associated with SZ.

ID	Description	*P*-value
hsa04964	Proximal tubule bicarbonate reclamation	.0034
hsa04260	Cardiac muscle contraction	.0044
hsa00790	Folate biosynthesis	.0047
hsa04925	Aldosterone synthesis and secretion	.0061
hsa04960	Aldosterone-regulated sodium reabsorption	.0092

KEGG analysis of DEGs genes of SZ (Top 5).

DEGs = differentially expressed genes, KEGG = kyoto encyclopedia of genes and genomes, SZ = schizophrenia.

### 3.3. *WGCNA network construction and identification of key* modules

**Figure 2.**
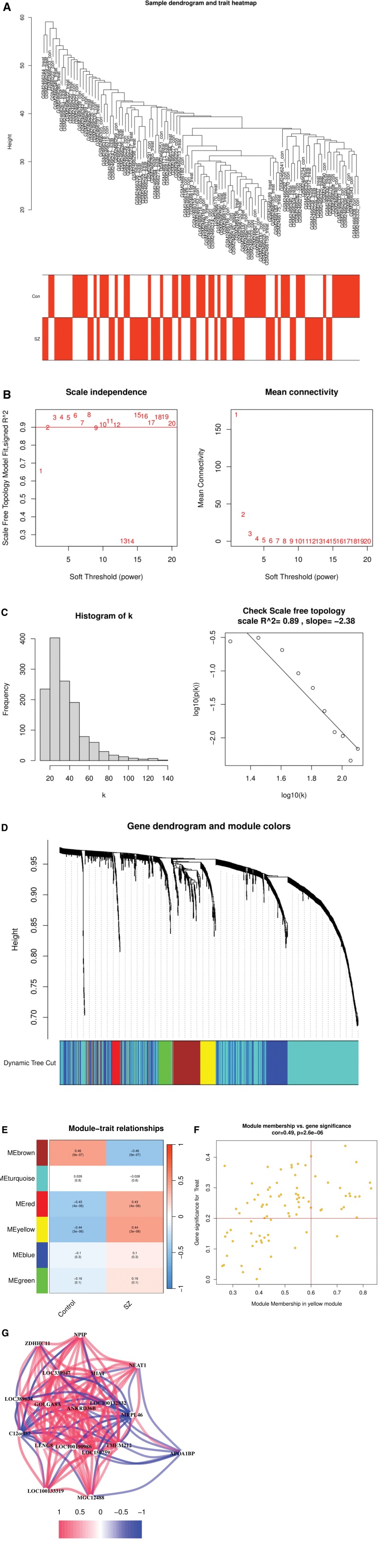
(A) The hierarchical clustering dendrogram and trait heatmap showed a clear clustering of SZ and control samples, confirming alignment with trait classification. (B) The determination of the soft threshold power for scale-free network construction was depicted. (C) The assessment of network scale-free topology present with a high R² value (0.89) and slope (−2.38), confirming the network satisfies scale-free criteria. (D) The gene dendrogram and module colors from hierarchical clustering was showed. (E) The module-trait relationship heatmap highlighted the “yellow” module’s strongest positive correlation with SZ (correlation = 0.44, *P* = 3 × 10^−6^). (F) A scatter plot of module membership versus gene significance in the “yellow” module showed a strong positive correlation (correlation = 0.49, *P* = 2.6 × 10^−6^) for SZ. (G) The correlation network of 18 hub genes was visualized in the “yellow” module, with red indicating positive correlations and blue indicating negative correlations. SZ = schizophrenia.

WGCNA was performed on combined datasets from SZ and control samples (GSE93987-1 and GSE93987-2) to identify the key genes associated with the SZ phenotype. Hierarchical clustering analysis confirmed clear sample clustering without outliers (Fig. 2A). A soft threshold power of 20 was selected to achieve a scale-free network topology with an R² value of 0.89, indicating a high network connectivity (Fig. 2B and C). 6 distinct gene modules with similar co-expression patterns were identified using hierarchical clustering (Fig. 2D). Correlation analysis of each module with the SZ phenotype identified the“yellow”module, consisting of 83 genes that were significantly associated with SZ. This module exhibited a correlation coefficient of 0.44 and a *P*-value of 3 × 10^−6^, demonstrating a robust relationship with SZ (Fig. 2E). Further analysis of the“yellow”module revealed a strong association between GS and MM, with a correlation coefficient of 0.49 and a *P*-value of 2.6 × 10^−6^ (Fig. 2F). The “yellow” module was designated as a critical module for SZ and included 18 hub genes. The expression levels of these hub genes were compared between the SZ and control groups and their interrelationships were analyzed using the “graph” package. The results revealed that APOA1BP displayed a notable negative correlation with other hub genes (Fig. 2G). These findings suggest that the “yellow” module and its hub genes, particularly APOA1BP, play a significant role in the molecular mechanisms underlying SZ.

### 3.4. Identification of key characteristic genes for SZ

**Figure 3.**
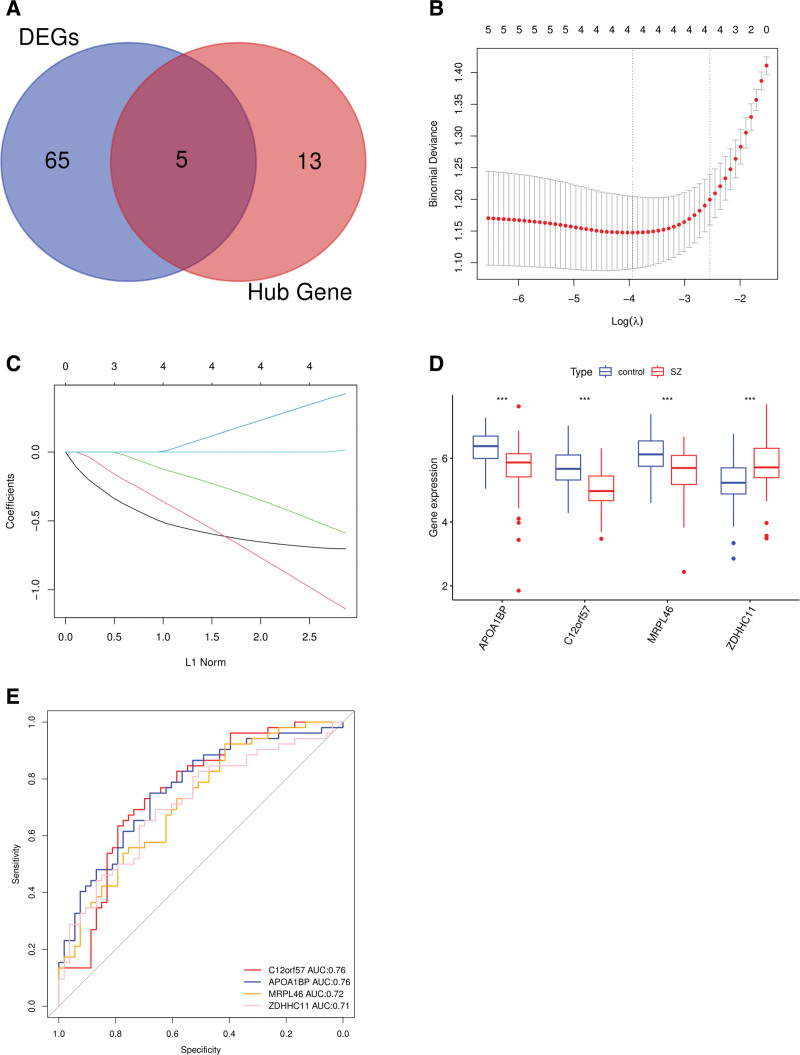
(A) Venn diagram showed the overlap of DEGs and hub genes identified by WGCNA. (B) LASSO regression model for SZ-associated genes displayed with binomial deviance plotted against log(λ), where the optimal λ value, minimizing deviance, was indicated by vertical dotted lines. (C) LASSO regression coefficient profiles showed SZ-related genes, highlighting 4 key genes (APOA1BP, C12orf57, MRPL46, and ZDHHC11) selected based on nonzero coefficients. (D) The expression levels of 4 genes was compared between SZ patients and controls, with *** indicating *P* <.001. (E) ROC curves for these4 genes, demonstrating their potential to distinguish SZ patients from controls. DEGs = differentially expressed genes, LASSO = least absolute shrinkage and selection operator, ROC = receiver operating characteristic, SZ = schizophrenia.

The intersection of the DEGs and hub genes identified 5 potential characteristic genes for SZ: APOA1BP, C12orf57, MRPL46, ZDHHC11, and MGC12488 (Fig. 3A). To further refine the selection of the characteristic genes, LASSO regression analysis was performed using lambda as the tuning parameter. This analysis identified 4 key genes, APOA1BP, C12orf57, MRPL46, and ZDHHC11, as the most representative biomarkers of SZ (Fig. 3B and C). The expression levels of these 4 genes were analyzed in 53 patients with SZ and 52 control subjects from the GSE93987 dataset (Fig. 3D). The results revealed that 3 genes (APOA1BP, C12orf57, and MRPL46) were significantly downregulated in patients with SZ (*P*<.001), while 1 gene (ZDHHC11) was significantly upregulated (*P*<.001).

ROC curve analysis was conducted to evaluate the diagnostic value of these genes in distinguishing patients with SZ from controls. As shown in Figure 3E, the curve (AUC) values for APOA1BP, C12orf57, MRPL46, and ZDHHC11 were 0.76, 0.76, 0.72, and 0.71, respectively, indicating their potential as diagnostic biomarkers for SZ.

### 3.5. Immune analysis and association with characteristic genes

**Figure 4.**
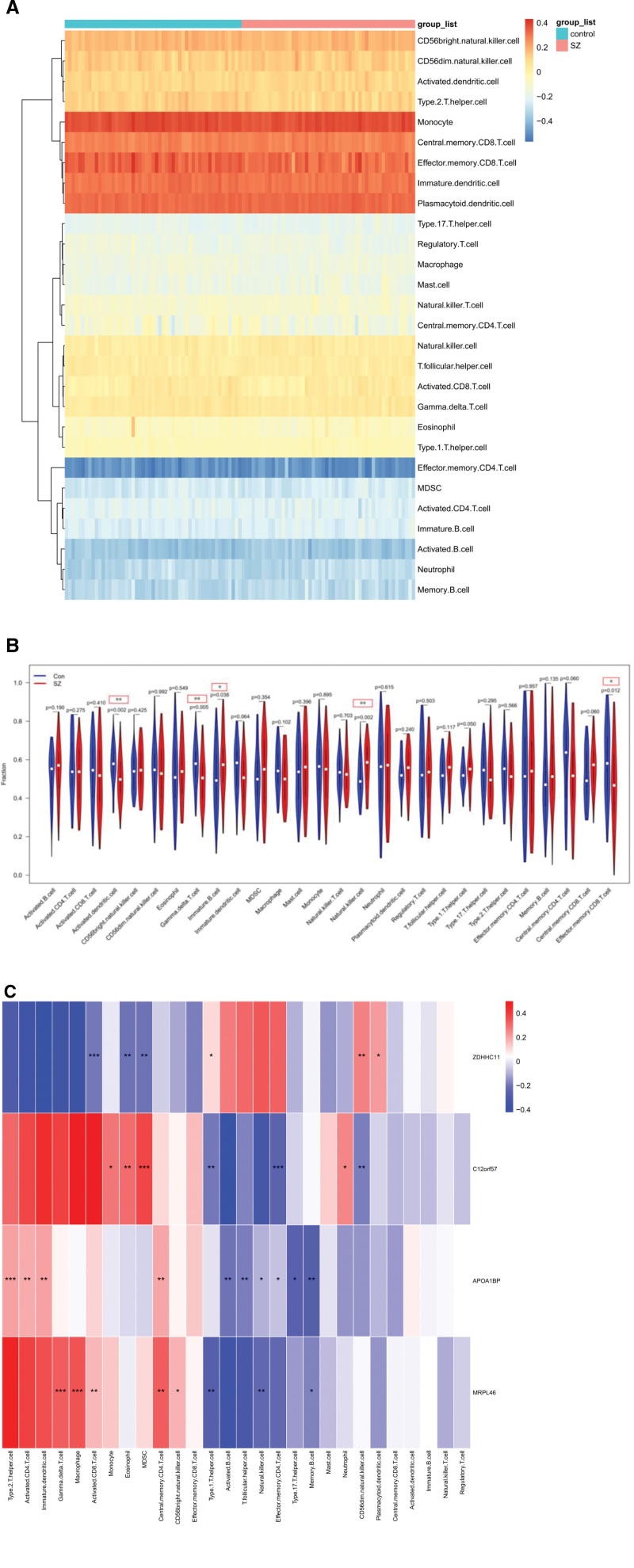
(A) Heatmap of the distribution of 28 immune cell types was showed in both groups. (B) Violin plot compared the infiltration levels of these immune cell types between SZ patients (red) and controls (blue). (C) Correlations was illustrated between key genes and immune cell infiltration levels in both groups, with red indicating positive correlations and blue indicating negative correlations. Significant associations are denoted by asterisks (*P <.05, **P <.01, ***P <.001). SZ = schizophrenia.

Differences in immune cell infiltration between patients with SZ and the controls were evaluated using ssGSEA. The distribution of the 28 immune cell types is shown in Fig.4A, where the horizontal axis represents sample identifiers and the vertical axis denotes immune cell categories. Hierarchical clustering of immune cell types is visualized along the vertical axis. Heatmap color intensity reflects the level of immune cell infiltration.

The analysis revealed that monocytes, plasmacytoid dendritic cells, effector memory CD8 T cells, central memory CD8 T cells, and immature dendritic cells were enriched in both the SZ and control samples. However, significant differences (*P* <.05) were observed for specific immune cell types. SZ samples showed enrichment in natural killer (NK) cells (*P* <.002) and immature B cells (*P* <.038), whereas activated dendritic cells (*P* <.002), gamma delta T cells (*P* <.005), and effector memory CD8 T cells (*P* <.012) were enriched in the controls (Fig. 4B). Correlation analysis between gene expression and immune cell infiltration (Fig.4C) demonstrated distinct associations: ZDHHC11 was positively correlated with CD56dim NK cells (*P* <.01) and negatively correlated with activated CD8 T cells (*P* <.001). C12orf57 was positively correlated with MDSCs (*P* <.001) and negatively correlated with effector memory CD4 + T cells (*P* <.001). APOA1BP was positively correlated with type 2 T helper cells (*P* <.001) but negatively correlated with memory B cells (*P* <.01). MRPL46 was positively correlated with macrophages (*P* <.001) and gamma delta T cells (*P* <.01), but negatively correlated with type 1 T helper cells and NK cells (*P* <.01).

Among the immune cells, NK cells (*P* <.002) and immature B cells (*P* <.038) showed significant positive correlations with SZ, indicating their potential roles in SZ pathogenesis. Notably, NK cells were negatively correlated with APOA1BP (*P* <.05) and MRPL46 (*P* <.01), whereas no significant correlations were observed between NK cells and C12orf57 or ZDHHC11.

### 3.6. Validation of immune-related genes

**Figure 5.**
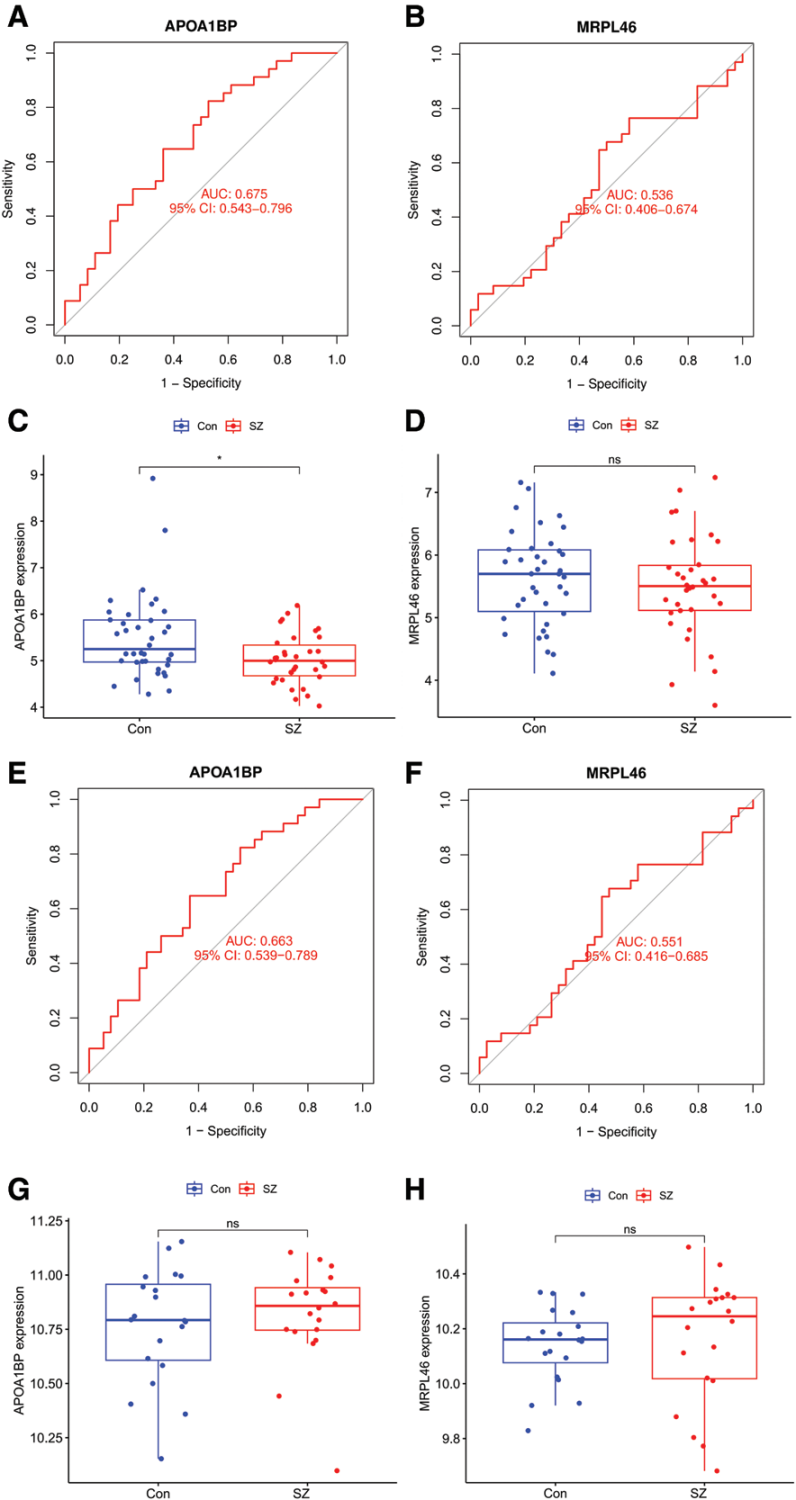
(A) and (B) ROC curve analyses for APOA1BP and MRPL46, respectively, were showed in the GSE87610 dataset. (C) and (D) Expression levels of APOA1BP and MRPL46 between SZ patients (red) and controls (blue) were showed in the GSE87610 dataset. (E) and (F) ROC curve analyses for APOA1BP and MRPL46 were showed in the GSE73129 dataset, (G) and (H) Expression levels between SZ patients and controls were displayed in the GSE73129 dataset. Statistical significance is indicated by *P <.05, and “ns” denotes no significant difference. ROC = receiver operating characteristic, SZ = schizophrenia.

To validate the findings from the training cohort, the diagnostic performance and expression levels of immune-related characteristic genes APOA1BP and MRPL46 were assessed in the testing cohorts (GSE87610 and GSE73129). In the GSE87610 dataset (34 SZ cases vs. 36 controls), ROC curve analysis revealed an area under the curve (AUC) value of 0.675 for APOA1BP and 0.536 for MRPL46, indicating that APOA1BP has greater diagnostic potential than MRPL46 (Fig. 5A and B). Expression analysis demonstrated that both genes were downregulated in SZ samples; however, only APOA1BP showed a statistically significant differential expression (*P* <.05; Fig. 5C and D). In the GSE73129 dataset (20 SZ cases vs 20 controls), the AUC value was 0.663 for APOA1BP and 0.551 for MRPL46, suggesting limited diagnostic performance in this cohort (Fig. 5E and F). Furthermore, neither APOA1BP nor MRPL46 showed significant differential expression between patients with SZ and controls in this dataset (Fig. 5G and H).

## 4. Discussion

Schizophrenia (SZ) is a neurodevelopmental disorder characterized by significant cognitive deficits, particularly in working memory (WM), which often emerge prior to clinical onset.^[23]^ Patients with SZ show reduced WM capacity compared to healthy individuals, with a progressive decline during adolescence.^[24]^ Functional and structural disruptions in regions such as the cortex, thalamus, and striatum during WM tasks suggest widespread impairments in cognitive networks.^[25–27]^ Postmortem investigations have uncovered a robust association between WM impairments in schizophrenia and irregularities in the 3-layered pyramidal neurons of the dorsolateral prefrontal cortex, a region essential for cognitive processing.^[28,29]^ Augmented DLPFC activation in humans and primates correlates with improved WM performance, underscoring the significance of microcircuitry involving pyramidal and inhibitory basket cells in preserving WM stability.^[30]^ Investigating molecular and pathological changes within the neural circuits of 3-layered pyramidal cells may uncover the mechanisms underlying cognitive impairment in SZ.

Although cross-dataset analyses require careful consideration of inherent heterogeneity among datasets – such as differences in sample processing protocols and microarray platforms, the results of PCAclearly demonstrated that batch effects were effectively mitigated. This strongly supports the high consistency and comparability of gene expression profiles across datasets. Moreover, tissue specificity may significantly influence the gene expression characteristics observed in different datasets. The GSE93987 was derived from postmortem DLPFC of patients with SZ, while the validation dataset GSE87610 was also obtained from postmortem DLPFC samples of SZ patients. In contrast, GSE73129 was derived from OE cells collected from living SZ patients. Although inter-tissue differences in gene expression may introduce bias in the identification of DEGs, DEGs identified in GSE93987 were consistently validated in GSE87610 with *P* <.05, but failed to demonstrate significant differential expression in GSE73129 (*P* >.05). In this study,70 DEGs were identified in the training cohort. GO analysis revealed that these DEGs were primarily involved in cellular communication via electrical coupling during cardiac conduction and telencephalon development, emphasizing their critical roles in brain development. These findings support the classification of SZ as a neurodevelopmental disorder, where early disruptions in neural development contribute to cognitive impairment and clinical manifestations.^[31–33]^ KEGG pathway analysis highlighted significant enrichment of pathways related to proximal tubule bicarbonate reclamation, cardiac muscle contraction, and folate biosynthesis.^[34,35]^ These findings suggest that targeting folate-related pathways may offer a therapeutic strategy for mitigating SZ symptoms, particularly in cognitive domains. In the training cohort, bioinformatics analysis identified APOA1BP and MRPL46 as key SZ-associated genes with reduced expression in the 3-layered pyramidal cells of DLPFC. Using ssGSEA, NK Cells and immature B cells were positively correlated with SZ in the DLPFC microenvironment, whereas APOA1BP and MRPL46 showed a negative correlation with these immune cell types. The validation cohort included 2 distinct tissues: postmortem DLPFC and neuronal layers from the OE of living SZ patients. The inherent differences between these tissues may influence the results, as the DLPFC is a central region for SZ-related changes, whereas the OE provides a peripheral, accessible source for neuronal assessment. Despite this, APOA1BP has emerged as a consistent gene of interest linked to immune responses in both tissues. These findings suggest that elevated NK cells in the DLPFC microenvironment coupled with altered APOA1BP expression may contribute to SZ pathogenesis. We acknowledge that tissue-specific heterogeneity across datasets may influence gene expression profiles and limit generalisability. Indeed, APOA1BP demonstrated consistent downregulation in postmortem DLPFC cohorts (GSE93987 and GSE87610) but not in the OE cohort (GSE73129). While this discrepancy highlights the potential variability introduced by different tissue origins, it also underscores the importance of DLPFC and OE tissues in exploratory analyses. Our approach was designed to first establish reproducibility within the same brain region and then extend evaluation to peripheral neuronal tissue, thereby balancing biological relevance with translational exploration. Nevertheless, larger, multi-tissue, and prospective cohorts will be required in future studies to validate and generalize these findings.

- APOA1BP, also known as NAXE, encodes an enzyme with dual functions critical for cellular homeostasis.^[36]^ Clinical observations of APOA1BP deficiency, particularly in pediatric populations, reveal severe neurological and systemic manifestations, including ataxia, cognitive decline, motor disturbances, psychiatric disorders, and pellagra-like skin lesions.^[37–39]^ In this study, the GSE98937 dataset revealed APOA1BP as a significantly down-expressed DEG with diagnostic potential for SZ (AUC = 0.76). Subsequent validation through WGCNA and LASSO regression analysis further confirmed its robust association with SZ pathogenesis. Importantly, independent validation in the GSE87610 cohort demonstrated conserved downregulated expression of APOA1BP (AUC = 0.675, *P* <.05), collectively establishing its molecular diagnostic value for SZ. We observed variability in the diagnostic performance of APOA1BP and MRPL46 across different cohorts. Several factors may account for these discrepancies. First, cohort heterogeneity – including tissue type (DLPFC vs. OE), may influence gene expression patterns and diagnostic outcomes. Second, technical variability across microarray platforms, despite batch-effect correction, could introduce residual differences. Emerging evidence increasingly supports a robust association between SZ and immune-related biomarkers. For example, Exogenous inflammatory stimuli selectively impair neural circuits governing motivational regulation and emotional processing. Chronic inflammatory responses induce progressive decoupling of functional connectivity within prefrontal-limbic networks, mechanistically contributing to treatment-resistant phenotypes in neuropsychiatric disorders^[40]^;In a Mendelian randomization analysis of European adult populations comprising over 30,000 SZ cases and 45,000 controls, genetically predicted C-reactive protein levels demonstrated protective effects against SZ. Conversely, each 2-fold increment in genetically determined soluble interleukin-6 receptor concentration was associated with a 6% increased risk of SZ oneset^[41]^; 1 research reveals SZ shows sex-specific IL-6/TNF-α linked to symptoms, chronicity-associated IL-1s/IL-10, revealing neuroimmune mechanisms with phenotype stratification^[42]^;One study suggest that peripheral inflammation may drive neurocognitive impairment through cytokine-mediated neural circuit dysregulation, highlighting the potential of immunomodulatory strategies for cognitive remediation in SZ treatment paradigms^[43]^;Evidence implicates prenatal herpesvirus-triggered neuroimmune dysregulation in SZ involving perivascular macrophage-mediated peripheral-central signaling and persistent immune reprogramming, underscoring the need to map spatiotemporal immune-viral interactions for targeted therapeutic development.^[44]^ NK cells, known for their innate immune role, have been reported to exhibit increased infiltration and activity in SZ, potentially exacerbating neuroinflammation and contributing to cognitive impairment.^[45,46]^ Similarly, 1 study identified 9 immune-linked hub genes and 10 dysregulated immune cell subtypes(NK cells, etc) in SZ, revealing novel therapeutic targets for SZ-associated immune dysregulation based on the analysis of the integrated multi-omics analysis (WGCNA/ssGSEA).^[47]^ Building on the previous findings, our study provides compelling evidence of a significant inverse correlation between APOA1BP expression and NK cell abundance in the DLPFC of SZ patients. The elevated presence of NK cells, coupled with their potential role in exacerbating neuroinflammation, strongly suggests that dysregulated APOA1BP expression contributes to a pro-inflammatory state. This, in turn, may amplify the neural and cognitive dysfunctions commonly observed in SZ. The redistribution of NK cells to prefrontal regions further supports their active involvement in modulating the pathological processes underlying SZ. Our results emphasize a negative correlation between APOA1BP expression and NK cell infiltration in the DLPFC suggests a potential link between metabolic dysregulation and immune activation in SZ. APOA1BP is involved in NAD(P)HX repair and cellular redox balance, processes essential for neuronal integrity. Downregulation of APOA1BP may create a pro-inflammatory microenvironment that favors NK cell recruitment and activation. Conversely, NK cell–mediated cytotoxicity and cytokine release may exacerbate neuronal stress and further reduce APOA1BP expression. While these mechanisms are biologically plausible, our bioinformatics approach cannot determine causality; therefore, the observed associations should be considered correlative. The positive association between NK cells and SZ severity, particularly with respect to cognitive impairment and neural dysfunction in the DLPFC, underscores the central role of this brain region in SZ pathology. These findings suggest that both immune and metabolic pathways may serve as potential therapeutic targets for alleviating SZ-related symptoms.

However, despite the strengths of our study, several limitations warrant consideration. First, while our findings were validated in the GSE87610, the lack of consistency in the GSE73129 highlights the variability introduced by tissue-specific differences and cohort heterogeneity, indicating the need for larger, more diverse datasets to enhance the generalizability of these results. Second, our study predominantly relied on bioinformatics analyses without experimental validation. While our integrative approach provides valuable correlative insights, mechanistic confirmation is lacking. Future in vitro studies, such as manipulating APOA1BP expression in neuronal or glial cell cultures and assessing its effects on inflammatory signaling and NK cell recruitment, would help clarify causality. Complementary in vivo experiments, including animal models with APOA1BP modulation, could further evaluate the impact on cognitive function and immune infiltration in the prefrontal cortex. These experimental approaches will be critical to substantiate and extend our bioinformatics-based findings. Third, a limitation of this study is the absence of detailed clinical metadata in the publicly available GEO datasets. Information on antipsychotic medication exposure, comorbidities, and lifestyle factors was not uniformly provided and therefore could not be considered in the analysis. These factors are known to influence both gene expression and immune cell profiles and may partially contribute to the variability observed across cohorts. Future investigations using prospectively collected cohorts with comprehensive clinical annotation will be essential to control for such confounders and to more precisely delineate the biological contribution of APOA1BP and NK cells to SZ pathophysiology. Lastly, the focus of this study on the DLPFC, while crucial, may not fully capture the breadth of SZ-related neural and immune alterations across other brain regions, meanwhile, future studies should consider more diverse cohorts to improve the applicability of the results, especially regarding demographic variations that may impact immune cell profiles and gene expression.

## 5. Conclusion

This study highlights APOA1BP and NK cells as potential biomarkers for schizophrenia-associated cognitive impairment. Beyond their diagnostic promise, these findings point to several future directions. First, larger and clinically well-characterized cohorts are needed to confirm their diagnostic performance across diverse populations. Second, the development of peripheral biomarker assays could facilitate translation into clinical practice. Finally, mechanistic studies exploring the interplay between APOA1BP and NK cells may reveal novel therapeutic targets, offering opportunities for immunomodulatory or metabolic interventions in SZ.

## Declaration

**Conceptualization:** Xu You.

**Data curation:** Xu You, Hongming Liang, Huabin Yang.

**Formal analysis:** Rongzong Shi.

**Funding acquisition:** Xu You.

**Investigation:** Xu You, Hongming Liang.

**Methodology:** Xu You.

**Resources:** Huabin Yang.

**Supervision:** Xu You, Hongming Liang.

**Writing – original draft:** Xu You, Huabin Yang.
